# Salivary VEGF: a non-invasive angiogenic and lymphangiogenic proxy in head and neck cancer prognostication

**DOI:** 10.1186/1755-7682-2-12

**Published:** 2009-04-24

**Authors:** Tahwinder Upile, Waseem Jerjes, Panagiotis Kafas, Shash Hirani, Sandeep U Singh, Marcel Guyer, Melissa Bentley, Holger Sudhoff, Colin Hopper

**Affiliations:** 1 The Professorial Unit, Royal National Throat, Nose and Ear Hospital, London, UK; 2 Head & Neck Cancer Centre, University College London Hospital, London, UK; 3 Department of Surgery, University College London Medical School, London, UK; 4 Department of Oral Surgery and Radiology, School of Dentistry, Aristotle University, Thessalonica, Greece; 5 Department of Otolaryngology, Bielefeld University, Bielefeld, Germany

## Abstract

**Background:**

Saliva is an enriched milieu containing biologically active proteins, including growth factors and cytokines. The endothelial growth factor family of proteins is important for the development of blood and lymphatic vessels in a healthy individual but also can aide tumour growth.

The aim of this study is to develop an independent normative database of values of salivary VEGF in a healthy population and to test the hypothesis that values would be raised in the saliva of patients with oral cancer.

**Methods:**

Twenty-one participants (12 males and 9 females) of whom 14 were healthy and 7 had oral squamous cell carcinoma took part in this study.

An immunoassay was employed to quantify a range of specific vascular endothelial and lymphatic endothelial growth factors in various body fluid compartments (blood, saliva). This was correlated to tumour factors and patient outcomes.

**Results:**

The mean salivary levels and serum VEGF A_165 _levels were significantly correlated in the sample as a whole. Additionally, both saliva and serum VEGF A_165 _levels were significantly correlated with age. There were significant differences in the salivary and serum levels of the control group and the cancer group.

**Conclusion:**

We present independent normative data on the levels of endothelial growth factor in the saliva of a healthy control population. We also suggest the use of simple non-invasive tests in helping to predict head and neck tumour biology and outcomes.

## Background

Saliva is an enriched milieu containing biologically active proteins, including growth factors and cytokines [1]. The endothelial growth factor family of proteins is important for the development of blood and lymphatic vessels in a healthy individual but can also aide tumour growth. Head and neck cancers are known to secrete high levels of endothelial growth factors which may aid their growth (angiogenesis) and metastasis (lymphangiogensis).

Vascular endothelial growth factor (VEGF or VEGF-A), also known as vascular permeability factor (VPF), is known for its important roles in regulating both physiological and pathological blood vessel growth. It is a member of the VEGF family that also includes VEGF-B, -C, -D, -E, and PlGF (placental growth factor). VEGF165 appears to be the most abundant isoform.

VEGF transcription is potentiated in response to hypoxia, oncogenic transformation, and growth factors. Upregulation of VEGF during conditions of low oxygen tension is dependent upon the activities of the hypoxia-inducible transcription factors Hif-1 and Hif-2. VEGF is a potent endothelial cell mitogen that can activate phospholipase C and induce rapid increases of free cytosolic Ca^2+^. VEGF has been shown to stimulate von Willebrand factor release from endothelial cells and induce expression of tissue factor in endothelial cells and monocytes. VEGF has also been shown to be chemotactic for monocytes and osteoblasts [2].

*In vivo*, VEGF can induce angiogenesis and increase microvascular permeability. As a vascular permeability factor, VEGF acts directly on the endothelium and does not degranulate mast cells. It promotes extravasation of plasma fibrinogen, leading to fibrin deposition which alters the tumor extracellular matrix. The modified extracellular matrix subsequently promotes the migration of macrophages, fibroblasts and endothelial cells. Based on its *in vitro *and *in vivo *properties, VEGF is expected to play important roles in inflammation and normal and pathological angiogenesis, a process that is associated with wound healing, embryonic development, and growth and metastasis of solid tumors [2,3].

Saliva is a known source of VEGF. The existence of a "salivary VEGF system" suggests that salivary VEGF plays a role in regulating physiologic and pathologic angiogenic and other vascular responses in salivary and mucosal tissues. In particular, the presence of VEGF in saliva may contribute to the remarkable healing capacity of the oral mucosa as well as other regions of the digestive tract [1]. The presence of considerable quantities of VEGF in normal human saliva suggests an important role for this cytokine in the maintenance of mucous membrane homeostasis. Rapid induction of neoangiogenesis by salivary VEGF helps accelerate the wound healing within the oral cavity. Moreover, salivary VEGF may permeabilize intraglandular capillaries and thus participate in the regulation of saliva production itself [3]. VEGF could be relevant to angiogenic processes in healthy as well as diseased periodontal tissue and that the periodontal status influences the salivary level of VEGF [4].

Many head and neck cancers are aerodigestive in origin, with the initial tumour developing from the epidermis exposed to carcinogenic agents. As the epidermal tumour develops the surface integrity is interrupted with exposure of the tumour to the digestive tract. The mucosal surface of these tumours is in direct contact with saliva and upper respiratory tract secretions and may contribute to salivary VEGF. Furthermore, tumour cells may secrete VEGF in an autocrine and paracrine fashion. It is hypothesized that the salivary VEGF content of these patients may yield as much prognostic information about the primary tumour as the serum VEGF. This would avoid the effects of other sources of VEGF such as tissue or blood factors. However the ulcerated acute and chronic inflammatory processes with superimposed infection and immune responses gives an elevated VEGF level that is not correlated just with the tumour but with the effects of the ulcerated and infected mucosa. This may be in itself pro-carcinogenic. Salivary VEGF levels seem to be associated with ulcer development in major recurrent aphthous ulceration [5,6].

Following the paucity of publications found during PubMed & Medline search using the key words "saliva", "VEGF2 and "cancer", we present the first independent normative database of values of salivary VEGF in a healthy population and test the hypothesis that values may be raised in the saliva of patients with oral cancer.

## Materials and methods

Twenty-one participants (12 males and 9 females) of whom 14 were healthy and 7 had oral squamous cell carcinoma (OSCC) took part in this study at the Royal National Throat, Nose & Ear Hospital and the Eastman Dental Hospital, London; the gender proportion in each group was similar with a mean age of 33.57 years. The protocol was approved by the Local Committee of the Ethics for Human Research.

An information sheet explaining the aim of our study in simple non-scientific terms was given to each of the patients. Each patient was asked to sign a consent form prior to the trial.

An immunoassay was then employed to quantify a range of specific vascular endothelial and lymphatic endothelial growth factors in various body fluid compartments (blood, saliva).

The subjects were asked to adequately hydrate in the 24 hours prior to sampling. Dehydrating agents such as caffeine and alcohol were avoided. Subjects were asked to swill out their oral cavity with water before whole saliva was collected using an AccuSorb™ (without enzyme binding or filtering capabilities) and assays were performed immediately. Serum was collected from venous puncture and the sample placed in a serum separator tube and allowed to clot for 30 minutes before centrifugation for 15 minutes at approximately 1000 × g. The serum was then assayed immediately.

VEGF_165 _was considered in particular since it is the most known and studied isoform in the current literature. We used the R&D Systems Human VEGF Immunoassay, QuantiGlo Human VEGF Immunoassay (Catalog Number QVE00B). The QuantiGlo Chemiluminescent VEGF Immunoassay is a 5.5 hour solid phase ELISA designed to measure VEGF_165 _levels in serum and saliva. This assay employs the quantitative sandwich enzyme immunoassay technique. A monoclonal antibody specific for VEGF has been pre-coated onto a microplate. Standards and samples were pipetted into the wells and any VEGF present was bound by the immobilized antibody. After washing away any unbound substances, an enzyme-linked polyclonal antibody specific for VEGF was added to the wells. Following a wash to remove any unbound antibody-enzyme reagent, an enhanced luminol/peroxide substrate solution was added to the wells and light was produced in proportion to the amount of VEGF bound in the initial step. A microplate luminometer (Specta Max 340 pc Molecular devices microplate reader) was used to measure the intensity of the light emitted. All samples were analysed in triplicate in independent experiments and representative data presented.

### Statistical analysis

Differences between groups were tested using the c2 test for frequency data and ANOVA/ANCOVA/Mann Whitney U Tests for continuous data. Where appropriate, measures of the strength of association between variables were provided through Cramer's phi (fc) and omega squared (ω^2^); add in interpretations guidelines for r^2 ^& ω^2^. Associations between variables were examined using Pearson's correlation coefficient, with the coefficient of determination (r^2^) as a measure of the strength of association. Significance levels for all tests were set at *p *< 0.05.

## Results

A direct correlation was made with T stage and N stage of OSCC patients and the levels of detectable endothelial growth factor. The effect of surgery was also determined.

There were 12 males and 9 females, with differing gender proportions in each group (Fisher's exact test p = 0.642; Φc = 0.204; control 7 male, 7 female: cancer 5 male 2 female OR although violation of assumption א2 = 0.875, df = 1, p = 0.350; Φc = 0.204). The mean age of the sample was 33.57 years (sd = 11.31), with the control group (mean = 28.714, sd = 10.126) significantly younger than the cancer group (mean = 43.286, sd = 6.264; F(1,19) = 12.004, p = 0.003, ω2 = 0.344).

**Figure 1:**
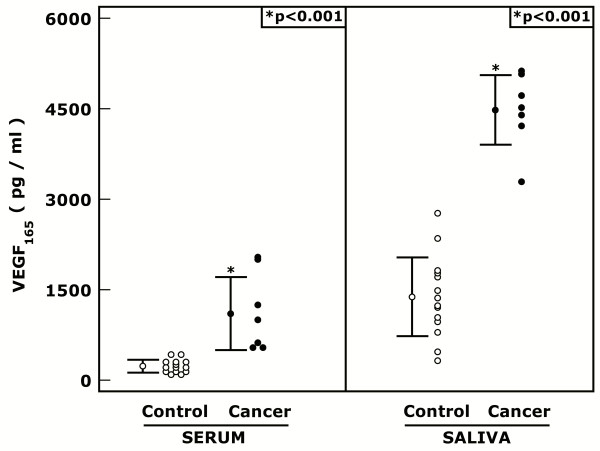
**Graph of Serum and Salivary VEGF A_165 _levels in control subjects and cancer patients**.

Mean individual specific saliva levels and serum VEGF A_165 _levels were significantly correlated (Pearson's r = 0.653, p = 0.001, r2 = 0.426), in the sample as a whole. The same relationship was calculated for each group separately (control: Pearson's r = 0.424, p = 0.131, r2 = 0.180; cancer: Pearson's r = -0.533, p = 0.198, r2 = 0.284) and did not significantly differ between the groups (z = 1.842, p = 0.066). Additionally, both saliva (Pearson's r = 0.584, p = 0.005, r2 = 0.341) and serum VEGF A_165 _levels (Pearson's r = 0.467, p = 0.033, r2 = 0.218) were significantly correlated with age (Figure 1).

The Mann-Whitney U test revealed significant differences: in the serum levels (U = 0, p < 0.001) of the control group (mean rank = 7.5) and the cancer group (mean rank = 18.0); and the saliva levels (U = 0, p < 0.001) of the control group (mean rank = 7.5) and the cancer group (mean rank = 18.0).

Furthermore, ANCOVAs examining treatment group differences revealed significant group effects on the serum measure (F(1,18) = 58.222, p < 0.001, ω2 = 0.337) after controlling for age (F(1,18) = 0.027, p = 0.871, ω2 ≈ 0.000), with the control group serum levels (mean = 1391.971 pg/ml, std. error = 199.364) significantly lower than the cancer group's (mean = 4451.005 pg/ml, std. error = 305.484); and significant group effects on the saliva measure (F(1,18) = 16.018, p = 0.001, ω2 = 0.491) after controlling for age (F(1,18) = 0.006, p = 0.938, ω2 ≈ 0.000), with the control group saliva levels (mean = 231.609 pg/ml, std. error = 113.970) significantly lower than the cancer group's (mean = 1148.875 pg/ml, std. error = 174.636). The homogeneity of regression slopes assumption was not violated in either analysis (Figure 1).

## Discussion

We present an independent normative data on the levels of endothelial growth factor in the saliva of a healthy control population. We also suggest the use of simple non-invasive tests in helping to predict head and neck tumour biology and outcomes [2-4].

The mean saliva levels and serum VEGF A_165 _levels were significantly correlated in the sample as a whole. Additionally, both saliva and serum VEGF A_165 _levels were significantly correlated with age. There were significant differences in the salivary and serum levels of the control group and the cancer group. Further, we found significant group effects on the salivary and serum measures of VEGF A_165 _after controlling for age with the control group salivary and serum levels significantly lower than the cancer group. Variations in the females of the control group were accounted for by the increased angiogenic activity during menstruation [7]. Differences between the control and cancer groups could not be accounted for by simple differences in gender proportions or age. We are collecting data on the group effects on the salivary and serum measures of VEGF A_165 _after controlling for age and gender.

Blood VEGF A_165 _was a good predictor of disease state [2-4] and the effect of surgery, however blood VEGF assessment is still an invasive test.

Salivary VEGF A_165 _is also an indicator of tumour burden and may possibly be employed as a non-invasive test. Its utility as a relatively inexpensive screening test for oral cavity cancer awaits larger multi-centered studies. The relevance of this pilot study lies in its possible development as a prognostic test with utility in the development of an effective prevention program to control head and neck cancer [8]. When compared with other highly diagnostic salivary tumour biomarkers, the advantages of this simple ELISA over the laboratory based 'in vitro detection of cytotoxic effects of saliva by using the plating efficiency index' [9] lie mainly in the ease of collection, simplicity of use and speed of results of the former. Despite this both tests have significant merit and may in fact be complementary. In conclusion saliva appears to provide a rich source of information regarding the epithelium, especially during neoplastic transformation.

## Competing interests

The authors declare that they have no competing interests.

## Authors' contributions

TU, WJ, PK, SH, SUS, MG, MB, HS, CH: contributed to conception and design, carried out the literature research, clinical study and manuscript preparation. All authors read and approved the final manuscript.
